# Effectiveness of a collagen matrix seal and xenograft in alveolar ridge preservation: an experimental study in dogs

**DOI:** 10.1038/s41598-023-50370-3

**Published:** 2024-01-02

**Authors:** Hee-seung Han, Jung-Tae Lee, Seunghan Oh, Young-Dan Cho, Sungtae Kim

**Affiliations:** 1 Department of Periodontology, School of Dentistry and Dental Research Institute, Seoul National University and Seoul National University Dental Hospital, 101 Daehak-no, Jongno-gu, Seoul, 03080 Republic of Korea; 2 One-Stop Specialty Center, Seoul National University Dental Hospital, Seoul, Republic of Korea; 3 Department of Dental Biomaterials, The Institute of Biomaterial and Implant, School of Dentistry, Wonkwang University, Iksan, Republic of Korea

**Keywords:** Diseases, Health care, Medical research, Materials science

## Abstract

Majority of previous studies on alveolar ridge preservation (ARP) used collagen membranes as barrier membranes, and further evidence for ARP in dehiscent extraction sockets with a deproteinized bovine bone mineral (DBBM) and matrix is needed. The aim of this study is to assess the impact of non-cross linked collagen membranes (membrane) and crosslinked collagen matrices (matrix) on ARP using DBBM in extraction sockets with buccal dehiscence. In six mongrel dogs, the mesial roots of three mandibular premolars (P2, P3, and P4) were extracted 1 month after dehiscence defect induction. Two experimental groups were randomly assigned: (1) DBBM with a membrane (DBBM/membrane group) and (2) DBBM with a matrix (DBBM/matrix group). Three-dimensional (3D) volumetric, microcomputed tomography (μCT), and histologic analyses were performed to assess the ridge preservation. Both groups were effective to maintain the ridge width (*p* > 0.05), and the DBBM/matrix group showed more favorable soft tissue regeneration and bone quality in the histological analysis (*p* = 0.05). Based on these results, DBBM/matrix could be better choice for ARP in cases of buccal dehiscence defects.

## Introduction

Tooth extraction can cause horizontal and vertical bone losses. A systematic review found that tooth extraction can cause horizontal bone loss of 29–63% or 3.79 mm and vertical bone loss of 11–22% or 1.24 mm after 6 months^1^. In cases of severely inflamed tooth extraction, bone loss may be more delayed, incomplete, and extensive^2,3^.

Alveolar ridge preservation (ARP) refers surgical interventions designed to maintain the initial shape of the alveolar ridge to the greatest extent possible, thereby mitigating the process of alveolar bone resorption^4^ Typically, ARP denotes a procedure involving the placement of bone graft material within the extraction socket, with or without the application of a covering barrier membrane^4^. The objective of ARP is to mitigate alterations in the alveolar ridge following tooth extraction, facilitate the generation of new bone within the extraction socket, and foster soft tissue healing at the entrance of the extraction socket^4^.

The ARP procedure can help compromise the extraction sockets and reduce bone loss^5^. A recent systematic review found that alveolar ridge preservation (ARP) with a compromised buccal wall reduced horizontal bone loss by 2.37 mm and vertical bone loos by 1.10 mm^6^. Typically, a collagen membrane is utilized to induce bone regeneration by covering the extraction socket and the top of the defect in these scenarios as part of the socket seal technique^7–9^. Both animal studies and clinical trials have demonstrated that socket seal surgery can increase bone regeneration, decrease loss of soft tissue, and stabilize the grafted biomaterial^10–12^.

According to a recent meta-analysis, both primary closure and ARP techniques, such as flap advancement and open healing with a barrier, can effectively reduce bone remodeling after tooth extraction^13^. Various types of barrier membranes, including dense-polytetrafluoroethylene (d-PTFE)^14,15^, collagen plug^16^, and collagen membrane^17–19^, can be used successfully in open healing with barrier technique for ARP procedures. Clinicians may choose a particular graft material based on their preference, funding, or cultural background. Absorbable collagen membranes are commonly used for ARP among the available barrier membranes. Additionally, previous studies have shown that the collagen barrier plays an osteoconductive role in supporting the growth of bone tissue^20,21^.

The collagen matrix has been used in clinical practice based on research indicating that maintaining soft tissue integrity over the area of ARP has a significant positive effect on bone healing. Collagen matrix is widely used as an alternative in gingival graft procedures for soft tissue augmentation^22,23^. collagen matrix with its two layers is thicker than the collagen membranes used for ARP^24,25^. Collagen matrix has shown that it facilitates blood clot stabilization, promotes tissue integration with one porous layer, and accommodates soft tissue healing with the other layers^26–28^. Depending on these characteristics, the collagen matrix could be considered as an alternative to barrier membranes for ARP^28–31^.

Collagen barrier membrane can be categorized into two types based on their crosslinking status: crosslinked and non-crosslinked. Studies indicate that cross-linking the collagen barrier membrane can decelerate absorption^32^, preserving the transplanted bone volume over an extended period^33^. However, there are reports suggesting that early exposure of the membrane may elevate complications related to soft tissue^34^.

Most of the aforementioned studies examining the use of the collagen matrices have focused on non-crosslinked collagen matrices. However, there are still insufficient data on the behavior and integration of the cross-linked collagen matrix (matrix). To address this gap, this study assessed the volumetric, radiographic, and histologic outcomes of the matrix and compared them to those of non-cross linked collagen membranes (membrane).

The underlying hypothesis of this study posits that the performance of ARP on teeth with buccal dehiscence defect will result in better maintenance of alveolar ridge width when a collagen membrane is utilized compared to cases where a collagen matrix is employed. The present study focuses specifically on the alveolar ridge of the extraction socket, with a primary emphasis on the buccolingual dimension of the ridge in areas exhibiting buccal dehiscence. The primary outcome measure involves the three-dimensional (3D) volumetric changes within the soft and hard tissues, along with the buccolingual width of the alveolar ridge.

## Results

### Clinical findings

**Figure 1:**
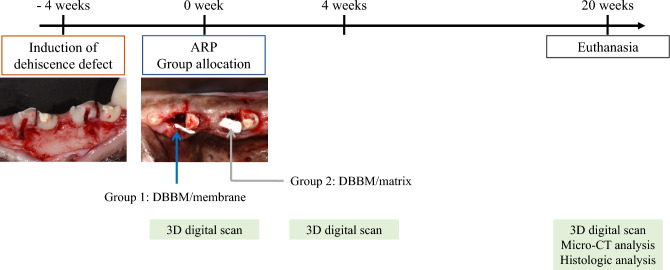
Study flowchart depicting the different stages of the experiment. DBBM: bovine-derived xenograft (Bio-Oss﻿®), membrane: type I non-crosslinked collagen derived from pigs (Bio-Gide﻿®), and matrix: type I crosslinked collagen derived from pigs (Collagen Graft 2﻿®).

After induction of the dehiscent defect, clinical signs of inflammation, namely gingival swelling, redness, and calculus deposition, were observed around the mesial root. Healing after the surgical procedure occurred without primary closure, but there were no signs of inflammation or other complications. A total of 24 sections were considered for 3D volumetric, radiographic, and histological analyses, with 12 sections in each group (Fig. 1).

### Analyses of the 3D digital images

**Figure 2:**
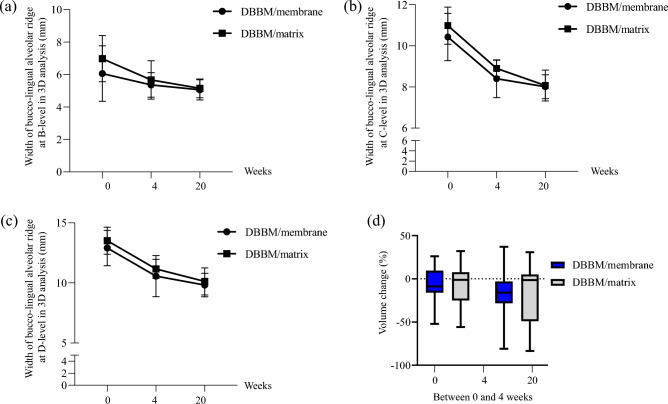
Results of repeated measures ANOVA of the linear and volumetric measurements (**a**–**d**) in both groups (DBBM/membrane and DBBM/matrix). (**a**–**c**) Buccolingual alveolar ridge width (mm) at 1 (B-level), 3 (C-level), and 5 (D-level) mm below the reference line over time. (**d**) Volume changes (%) in the volume of interest (VOI) between 0 and 4 weeks and between 0 and 20 weeks. DBBM: bovine-derived xenograft (Bio-Oss﻿®), membrane: type I non-crosslinked collagen derived from pigs (Bio-Gide®﻿), and matrix: type I crosslinked collagen derived from pigs (Collagen Graft 2﻿®﻿).

The widths is shown in Fig. 2 and Table S1. The change over time was significant within both groups as a result of repeated-measures ANOVA at 1, 3, and 5 mm below the crest (*p* < 0.001), and was used as a linear model (*p* < 0.001).

Linear changes in the membrane and matrix were not statistically significant at 1, 3, and 5 mm below the crest (Table S1). Volumetric measurements of the volume of interest (VOI) are shown in Fig. 2 and Table S1. The volume change ratios of DBBM/membrane and DBBM/matrix were statistically insignificant.

### Analyses of micro-CT radiographic images

The results of the qualitative and quantitative analyses of the micro-CT images are presented in Table 1. The BV/TV values were 32.53 ± 3.93 and 32.42 ± 3.24% in the DBBM/membrane and DBBM/matrix group, respectively. The BS/BV values were 6.727 ± 2.10 and 6.296 ± 1.94, in the DBBM/membrane and DBBM/matrix groups, respectively. The TbPf values, which indicate the level of interconnectivity of the bone, were − 6.31 ± 3.19 and − 6.08 ± 2.96 in the DBBM/membrane and DBBM/matrix groups, respectively. SMI, similar to TbPf, displayed no significant differences; thus, no significant qualitative differences were observed between the two groups.

**Table 1:** Qualitative and quantitative micro-computed tomography (μCT) analyses at 20 weeks of healing.

	Qualitative analysis						Quanitative analysis		
	BV (mm^3^)	TV (mm^3^)	BV/TV (%)	BS/BV (mm ^−1^)	TbPf	SMI	Coronal (%)	Middle (%)	Apical (%)
DBBM/membrane	444.55 ± 208.98	1363.41 ± 590.73 (median: 1441.816)	$32.53 \hspace{0.17em}\pm \hspace{0.17em} 3.93$	$6.727\hspace{0.17em}\pm \hspace{0.17em}2.10$ (median: 6.022)	$-6.31\hspace{0.17em}\pm \hspace{0.17em}3.19$	$-5.82\hspace{0.17em}\pm \hspace{0.17em}3.23$	$27.47\hspace{0.17em}\pm \hspace{0.17em}17.15$	$78.65\hspace{0.17em}\pm \hspace{0.17em}14.64$	$92.08\hspace{0.17em}\pm \hspace{0.17em}4.49$ (median: 93.20)
DBBM/matrix	402.00 ± 179.17	1234.01 ± 384.10 (median: 1351.917)	$32.42\hspace{0.17em}\pm \hspace{0.17em}3.24$	$6.296\hspace{0.17em}\pm \hspace{0.17em}1.94$ (median: 5.572)	$-6.08\hspace{0.17em}\pm \hspace{0.17em}2.96$	$-5.95\hspace{0.17em}\pm \hspace{0.17em}3.10$	$29.53\hspace{0.17em}\pm \hspace{0.17em}20.60$	$79.86\hspace{0.17em}\pm \hspace{0.17em}5.65$	$92.42\hspace{0.17em}\pm \hspace{0.17em}4.37$ (median: 93.08)
*p* value	0.555	0.920	0.941	0.514	0.855	*0.917*	0.776	0.793	0.852

*BV* Bone volume, *TV* Total volume, *BS* Bone surface, *TbPf*, trabecular bone pattern factor, *SMI* structure model index.

DBBM: Bio-Oss^®^, membrane: Bio-Gide^®^, matrix: Collagen Graft 2^®^.

The dimensional proportions at the coronal area were 27.47 ± 17.15 and 29.53 ± 20.60% in the DBBM/membrane and DBBM/matrix groups, respectively; there were no statistically significant differences between the two groups at the 20-week observation. The dimensions in the middle and apical areas were 78.65 ± 14.64 and 79.86 ± 5.65%, along with 92.08 ± 4.49 and 92.42 ± 4.37% in the DBBM/membrane and DBBM/matrix groups, respectively; there were no significant intergroup differences at the middle and apical areas.

### Analyses of histologic samples

**Figure 3:**
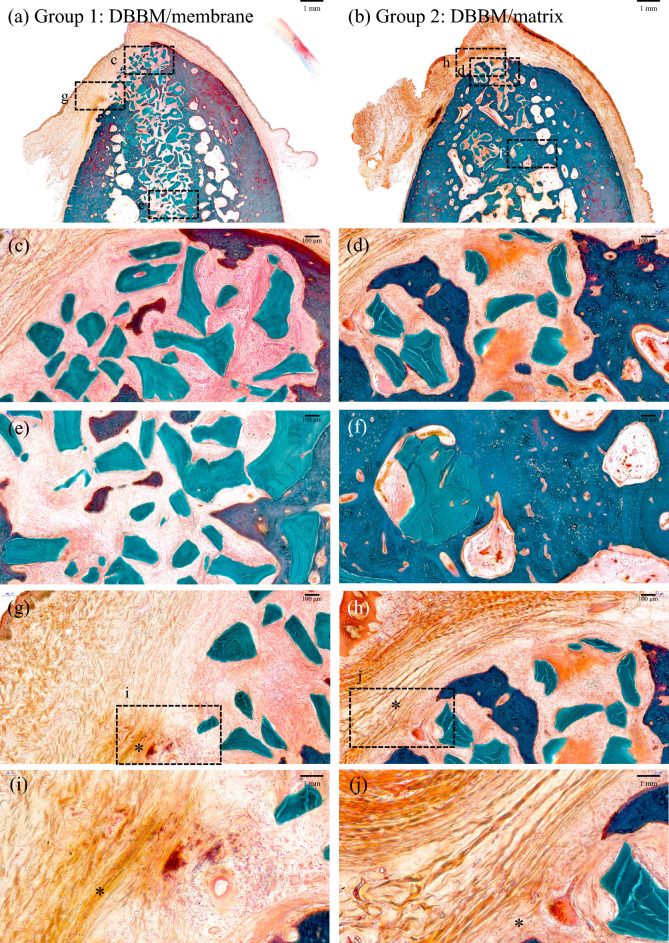
Buccolingual section obtained at 20 weeks after staining with Goldner's trichrome (**a**–**j**). (**a**, **b**) Low-magnification images of the membrane and matrix groups (scale bar = 1 mm). High-magnification images of (**c**, **d**) crestal, (**e**, **f**) apical, (**g**, **h**) soft tissue areas (scale bar = 100 um) are shown in panels (**a**, **b**), and high-magnification images of (**i**, **j**) pseudo-periosteum (scale bar = 50 um) are shown in panels (**g**–**h**), respectively. *: pseudo-periosteum. DBBM: bovine-derived xenograft (Bio-Oss﻿®), membrane: type I non-crosslinked collagen derived from pigs (Bio-Gide﻿®), and matrix: type I crosslinked collagen derived from pigs (Collagen Graft 2﻿®).

Following a 20-week period of socket healing, both groups exhibited robust healing of the extraction sockets characterized by the presence of a solid osseous structure interlaced with connective tissue (Fig. 3). In regions where DBBM was employed, there was a mixture of newly generated bone, vestiges of pre-existing bone particles, and connective tissue.

In the DBBM/membrane group, the augmented area remained well-preserved (Fig. 3B), and newly mineralized bone was observed in the coronal part of the sockets below the newly formed collagen matrix. In the coronal and apical areas of the extraction sockets, DBBM particles were dispersed in the connective tissue, and osteoclasts were present in their lacunae at the boundary (Fig. 3C). A pseudo-periosteum was observed along the outer surface of the alveolar ridge (Fig. 3G). The membrane was almost completely absorbed and integrated into the newly formed soft connective tissue. Clusters of red-stained fibers were embedded in and transversed by prominent newly formed collagen fiber bundles with interspersed slender fibroblasts. Blood vessels are also present in the collagen matrix.

In the DBBM/matrix group, the augmented area was well maintained and dome-shaped (Fig. 3B), with most DBBM particles tightly attached to the newly formed woven bone. Newly formed bone had pores, and osteoblast-like cells were observed on its surface. The DBBM particles were in contact with the surrounding mineralized bone rather than with the fibrovascular tissue (Fig. 3F). Blood vessels and a pseudo-periosteum were observed and the matrix was almost fully integrated into the newly formed soft connective tissue above the pseudo-periosteum (Fig. 3H). The matrix was mostly absorbed; however, there were areas where the matrix was occasionally observed. Clusters of red-stained fibers were embedded in and transversed by prominent newly formed collagen fiber bundles with interspersed slender fibroblasts. Blood vessels that sprouted into the matrix-stained light pink were also present in the new collagen matrix and pseudo-periosteum.

### Histomorphometric analyses

The width of the ridge at different points was not significantly different between the two groups (Table 2). Similarly, there were no significant differences in the amounts of mineralized bone, or fibrovascular connective tissue between the two groups, as determined by histomorphometry (Table 3). However, the number of residual bone particles was two times higher in the DBBM/membrane group, which was statistically significant.

**Table 2:** Width measurements (Mean ± SD) of buccal and lingual alveolar compartments of the ridge at three different levels: 0 mm (A), 1 mm (B), 3 mm (C), and 5 mm (D) below the alveolar crest.

Group	Buccal-median				Lingual-median			
	A	B	C	D	A	B	C	D
DBBM/membrane	1.49 ± 0.33	2.17 ± 0.37	3.05 ± 0.60	4.01 ± 0.69	1.17 ± 0.53	2.31 ± 0.44	3.51 ± 0.52	4.41 ± 0.64
DBBM/matrix	1.88 ± 0.67	2.45 ± 0.60	3.04 ± 0.74	3.98 ± 0.87	1.13 ± 0.36	2.35 ± 0.35	3.60 ± 0.43	4.57 ± 0.41
*p* value	0.081	0.182	0.671	0.514	0.845	0.804	0.644	0.475

DBBM: Bio-Oss^®^, membrane: Bio-Gide^®^, matrix: Collagen Graft 2^®^.

**Table 3:** Areal measurements (Mean ± SD) of histomorphometric analysis in ROI (%).

Group	Mineralized bone	Residual bone particle	Fibrovascular connective tissue
DBBM/membrane	45.34 ± 6.79	6.52 ± 4.85 (median: 5.538)	48.14 ± 7.65
DBBM/matrix	49.45 ± 8.50	3.20 ± 3.32 (median: 2.141)	47.06 ± 7.89
*p* value	0.204	0.05*	0.736

DBBM: Bio-Oss^®^, membrane: Bio-Gide^®^, matrix: Collagen Graft 2^®^

*Significantly different between two groups (Statistical significance level was 5%, *p* < 0.05).

## Discussion

In this study, we compared the effects of two ARP procedures, using DBBM with a membrane and DBBM with a matrix, in extraction sockets with buccal dehiscence in dogs. Crest height and width were markedly reduced following extraction and defect induction. Although no significant differences in the 3D volumetric and radiographic analyses were found, a significant difference was observed in the residual bone particles in the DBBM/membrane.

In this study, the upper portion of the socket underwent coverage with either a collagen membrane or collagen matrix, with open healing being deliberately induced. While open healing comes with the drawback of exposing the bone graft material to the oral environment during the initial stages of healing, it offers the benefit of reducing trauma^35^ and minimizing alveolar ridge absorption^19^.

The different properties and preparations of the membrane and matrix explain the different behaviors observed in this study. In this study, membranes (derived from porcine dermis and consisting of type I and III collagen) were non-crosslinked^36^, whereas the matrix (derived from porcine tendon and consisting of type I collagen) was crosslinked with 1-ethyl-3-(3-dimethylaminopropyl) carbodiimide hydrochloride (EDC) to improve resistance to enzymatic degradation. The amount of water that collagen can bind to depends on its crosslinking^37^. Non-crosslinked collagen binds more water than crosslinked collagen. The membrane swelled more initially, but degraded faster over time than the matrix^38^. The matrix exhibited more stable swelling and showed good results in a previous study^39^.

Collagen membrane resorption generally takes 4–12 weeks^40^, but the rate of absorption is influenced by the composition of the collagen membrane and local environmental factors such as pH and temperature^41^. Previous studies have shown that the structural properties of non-crosslinked collagen matrices are designed to maintain barrier function for at least 30 days^42,43^. However, in this study, a cross-linked collagen matrix was used, which was expected to maintain its barrier function for an even longer period, thereby holding the potential to enhance outcomes through the mitigation of grafting material collapse. The results of this study are similar to those of previous studies^31,44^ that used DBBM and a matrix for ARP, suggesting that the matrix could be useful for soft tissue reconstruction during ARP.

However, the lack of significant differences in the residual bone particles between the two groups requires cautious interpretation. Histomorphometric analysis of the DBBM/membrane and DBBM/matrix groups showed that the residual bone particles in the region of interest (ROI) is almost double, which indicates the need for further discussion concerning the large standard deviation of the DBBM/membrane group. The slightly higher proportion of residual bone particles in the DBBM/membrane group is consistent with previous studies that reported incomplete integration and reabsorption even after 24 months of DBBM^45^. Therefore, it is possible that a few residual bone materials may still exist even after sufficient time for delayed implant placement following ARP with DBBM/membrane.

While the utilization of various bone graft materials during ARP procedures often does not significantly enhance new bone formation compared to natural healing, employing DBBM with a collagen barrier in teeth with dehiscence demonstrates a noteworthy reduction in the future need for guided bone regeneration (GBR)^46^. Additionally, the execution of ARP results in diminished horizontal and vertical bone loss compared to cases where ARP is not performed^6^.

This study has some limitations. First, the absence of multiple time points for radiographic and histological analyses may have limited the interpretation of the results. Short-term observational periods could provide further insights into the healing process of extraction sockets with buccal dehiscence and the behavior of the membrane or matrix. Secondly, the relatively small dimensions of the defects could promote regeneration and limit the applicability of the data in clinical settings. Third, the lack of a negative control group without ARP after the induction of dehiscence should be considered.

Within the limitations of this study, the effects of volume change and bone quality and quantity in radiographic analysis were not statistically significant between the two groups; however, the DBBM/matrix showed slightly favorable histologic results. DBBM/matrix could be a candidate therapy for successful ridge regeneration in extraction sockets with buccal dehiscence defects.

## Materials and methods

### Animals

The study was conducted between August 2021 and February 2022 at the CRONEX Animal Facility in Seoul, Korea. Six male mongrel dogs, weighing 31–35 kg and aged 10–12 months, were housed in a cage with a constant temperature (22 ± 2 °C) and humidity (50 ± 10%). All experimental protocols were approved by the Institutional Animal Care and Use Committee of CRONEX (IACUC; approval no. 202108010) and carried out according to the guideline and regulations of IACUC of CRONEX, which adhered to the ARRIVE guidelines for pre-clinical studies^47^.

Based on the previous study^48,49^, the sample size was determined to minimize the number of animals in each group and to distribute the samples equally across each experimental site.

### Study design

The overall study flow was presented in Fig. 1. A buccal dehiscence defect model^50^ was constructed in order to simulate a damaged extraction socket (the experimental site). After inducing the dehiscence defect, the mesial roots of the mandibular second, third, and fourth premolars (P2, P3, and P4, respectively) were extracted 4 weeks later. Each distal root was subjected to root canal treatment after hemisection and was kept as a reference pristine site for the corresponding mesial root. The following two extraction sockets on the unilateral alveolar ridge were rotated into the following groups to produce an equal distribution of three other premolar sites:

DBBM/membrane group: ARP with DBBM (Bio-Oss^®^; Geistlich Pharma AG, Wolhusen, Switzerland) and a membrane (Bio-Gide^®^, Geistlich Pharma AG, Wolhusen, Switzerland)

DBBM/matrix group: ARP with DBBM (Bio-Oss^®^; Geistlich Pharma AG, Wolhusen, Switzerland) and a matrix (Collagen Graft 2^®^, Genoss, Suwon, Korea)

### Surgical procedures

All surgical procedures were performed under general anesthesia induced by a 1:1 mixture of tiletamine hydrochloride, zolazepam hydrochloride (2 mL/10 kg intravenously, Zoletil^®^ 50; Virbac S.A., France), and xylazine hydrochloride (Rompun^®^; Bayer, Leverkusen, Germany). Inhalation anesthesia was induced by the inhalation of a 2:1 mixture of isoflurane and oxygen. Local infiltration anesthesia (Lidocaine HCL 2% with epinephrine 1:100,000; Huons, Gyeonggi-do, Korea) was administered via infiltration at the buccal and lingual sides of teeth P2, P3, and P4. Scaling was performed prior to surgery. To manage postoperative infection and pain, enrofloxacine (0.2 mL/kg intramuscular, Komibiotril 100 Injection®; Komipharm Co. Ltd., Siheung-si, Korea) and meloxicam (0.4 mg/kg analgesics intramuscular, Metacam®; Labiana Life Science, S.A., Spain) were administered for 3 days. The oral mucosa and wound were disinfected seven times a week using a 0.12% chlorhexidine solution (Hexamedin®; Bukwang Pharm Co., Seoul, Korea).

### Induction of dehiscence defect

In this study, the surgical techniques were similar to those of a previous study^50^ except for the bone graft materials used and healing periods. This study involved hemisection of three premolars on one side of the mandible of six male mongrel dogs after flap elevation with an intracrevicular incision. The remaining distal root was subjected to root canal treatment and filled with gutta-perch and a sealer (AH Plus; Dentsply DeTrey, Konstanz, Germany), and the coronal access to the pulp chamber was sealed with intermediate restorative material (IRM; Dentsply Sirona, Milford, DE, USA). A dehiscent defect was created by performing osteotomy of the buccal wall at the mesial root and creating a groove on the buccal area to expose the dental pulp. The mesiodistal (MD) width of dehiscence was standardized to 2/3 of the MD width of the mesial root. Additionally, the height (H) of the dehiscence was established at half the H of the mesial root.

### Extractions and ridge preservation procedures

Following 4 weeks, the corresponding mesial roots were extracted and group-specific interventions were conducted. The DBBM was filled into the extraction socket up to an imaginary line between the adjacent bone walls. The membrane or matrix was trimmed into a rectangular shape to cover the top of the extraction socket, which is grafted area^48^. Open healing was induced by exposing the collagen membrane or collagen matrix without primary closure of the flap.

### Sacrifice

The animals were sacrificed 5 months^51^ after surgery using suxamethonium chloride hydrate (50 mg/mL intraveneously, Succipharm^®^; Komipharm Co. Ltd, Siheung-si, Korea) for radiographic and histologic analyses. The acquired specimens were fixed in 10% neutral buffered formalin for 10 days.

### Analyses of the 3D digital images

The mandibles of the dogs were scanned using a dental scanner (Medit, Seoul, Korea), and the obtained images were superimposed at 0, 4, and 20 weeks after surgery for 3D digital evaluation using software (Geomagic Design X and Control X, 3DSYSTEMS, SC, USA). Buccolingual alveolar ridge width was measured on cross-sectional images according to previous studies^52–54^. A reference line and curve were created, and three parallel lines (B, C, and D-line) were drawn to connect the buccal and lingual contours 1, 3, and 5 mm below the top of the alveolar crest at three time points. A rectangular area of 5 × 4 mm was selected as the region of interest (ROI) to measure volume changes during the three overlapping observational periods^55^.

### Analyses of micro-computed tomography radiographic images

Micro-CT (Skyscan 1173, Konitch, Belgium) was used to obtain the scan data at a resolution of 35 μm, and DataViewer (Skyscan, Konitch, Belgium) was used for visualization. Bone morphometric parameters were measured for each volume of interest (VOI), according to the American Society for Bone and Mineral Research Histomorphometry Nomenclature Committee^56,57^: bone volume (BV), tissue volume (TV), volume density (BV/TV), bone surface (BS), surface-to-volume ratio (BS/BV), trabecular bone pattern factor (TbPf), structure model index (SMI). Buccolingual sectioning was performed, and four horizontal lines were drawn to calculate the dimensional changes between the experimental and reference areas, expressed as percentages^9,58^.

### Analyses of histologic samples

The sectioned specimens were embedded in acrylic resin (Technovit 7200 VLC; Heraeus Kulzer, Wehrheim, Germany) and cut into 40 μm slices, then stained with Goldner’s trichrome stain and analyzed by an experienced investigator using a software (CaseViewer; 3DHISTECH Ltd., Budapest, Hungary, ImageJ; Bethesda, Maryland, USA). The landmarks were identified, and the buccolingual alveolar bone width was measured at four points: A, the crest of the buccolingual bone wall of the ridge; V, the vertical line drawn in the center of the ridge through the most apical point of the osseous basal body that separated the buccolingual compartments of the section; T, the line perpendicular to V drawn through the apical point of the basal bone; and B, C, and D, the lines perpendicular to V at 1, 3, and 5 mm below peak A^51^. An imaginary line was drawn from the alveolar bone crest to set the ROI. Surface area of the mineralized bone, residual bone particles, and fibrovascular connective tissue on the ROI were measured and calculated^9,59^. The investigator performed histomorphometry on 36 sections twice over 2 weeks to adjust for errors.

### Statistical analyses

Statistical analyses were performed using SPSS version 26.0. The data are presented as mean ± standard deviation, and normality and sphericity assumptions were tested using the Shapiro–Wilk and Mauchly’s tests, respectively. Changes between the two groups were compared using repeated-measures analysis of variance (ANOVA) with the Bonferroni correction. For intergroup comparisons, the Student's t-test or Mann–Whitney U test was used. The significance threshold was set at *p* < 0.05 The intraclass correlation coefficient (ICC) was used to assess examiner reliability, which was high for both radiographic and histomorphometric evaluations. The concordance coefficients were 0.984 and 0.93, respectively, indicating a high reliability.

### Ethical approval

This study was approved by the Institutional Animal Care and Use Committee of CRONEX, Seoul, Korea (approval No. 202108010) according to the ARRIVE guidelines for preclinical studies.

### Supplementary Information

Supplementary Figure S1.

Supplementary Table S1.

## Data Availability

All data generated by this study are included in this manuscript.
