# Supplementary material for: Effectiveness of a collagen matrix seal and xenograft in alveolar ridge preservation: an experimental study in dogs
Source: Sci Rep. 2024 Jan 2;14:163. doi: 10.1038/s41598-023-50370-3 (PMC10762190; doi:10.1038/s41598-023-50370-3)
Supplement: Supplementary file 1 — Supplementary Figure S1. [file 41598_2023_50370_MOESM1_ESM.pptx]

## Slide 1
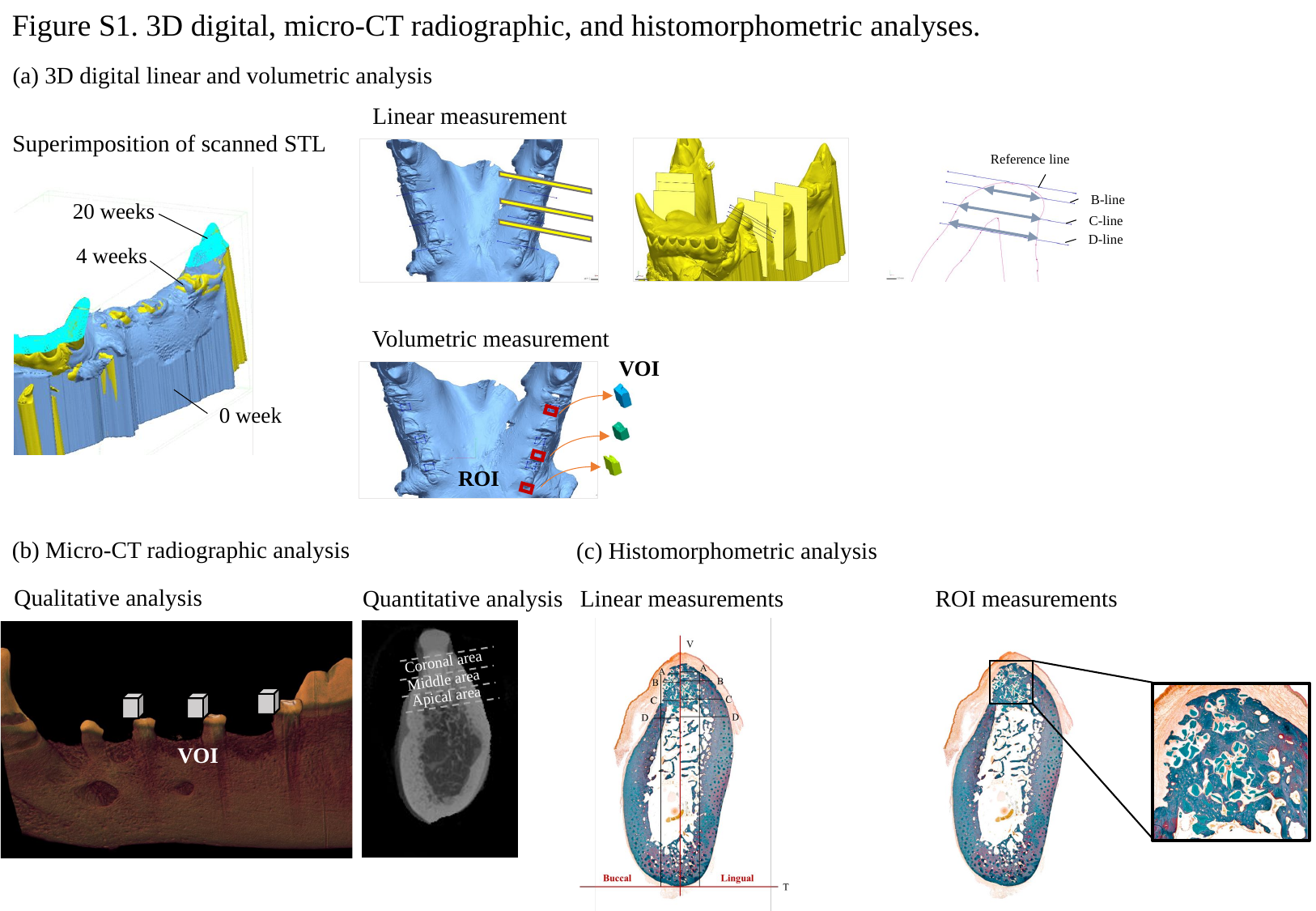

Figure S1. 3D digital, micro-CT radiographic, and histomorphometric analyses.
(a) 3D digital linear and volumetric analysis
Linear measurement
Superimposition of scanned STL
Reference line
B-line
C-line
D-line
20 weeks
4 weeks
0 week
Volumetric measurement
VOI
ROI
(b) Micro-CT radiographic analysis
(c) Histomorphometric analysis
Qualitative analysis
Quantitative analysis
ROI measurements
Linear measurements
Coronal area
Middle area
Apical area
VOI
