# Supplementary material for: Effectiveness of a collagen matrix seal and xenograft in alveolar ridge preservation: an experimental study in dogs
Source: Sci Rep. 2024 Jan 2;14:163. doi: 10.1038/s41598-023-50370-3 (PMC10762190; doi:10.1038/s41598-023-50370-3)
Supplement: Supplementary file 2 — Supplementary Table S1. [file 41598_2023_50370_MOESM2_ESM.docx]

**Effectiveness of a Collagen Matrix Seal and Xenograft in Alveolar Ridge Preservation: An experimental study in dogs**

**Supplementary Tables**

|  | Ridge width (mm) | | | | |  | Ridge width changes (mm) between | | |  | Volume changes (mm^3^) between | | |
| --- | --- | --- | --- | --- | --- | --- | --- | --- | --- | --- | --- | --- | --- |
|  | 0 week | | 4 weeks | | 20 weeks |  | 0 week and 4 weeks | 0 week and 20 weeks | |  | 0 week and 4 weeks | 0 week and 20 weeks | |
|  | DBBM/membrane group | | | | | | | | |  | -7.91 ± 21.28 | | -19.69 ± 31.15 |
| B-line | 6.06 ± 1.71 | 5.36 ± 0.76 | | 5.06 ± 0.62 | |  | -0.70 ± 2.14 | | -1.00 ± 1.88 |  |  | |  |
| C-line | 10.43 ± 1.15 | 8.40 ± 0.92 | | 8.01 ± 0.58 | |  | -2.04 ± 1.33 | | -2.42 ± 1.07 |  |  | |  |
| D-line | 12.91 ± 1.47 | 10.57 ± 1.71 | | 9.83 ± 0.98 | |  | -2.33 ± 1.75 | | -3.08 ± 0.77 |  |  | |  |
|  | DBBM/matrix group | | | | | | | | |  | -8.35 ± 26.80 | | -16.75 ± 35.43 |
| B-line | 6.98 ± 1.42 | 5.67 ± 1.18 | | 5.15 ± 0.58 | |  | -1.31 ± 1.59 | | -1.83 ± 1.54 |  |  | |  |
| C-line | 10.98 ± 0.90 | 8.90 ± 0.40 | | 8.07 ± 0.75 | |  | -2.07 ± 1.12 | | -2.91 ± 1.39 |  |  | |  |
| D-line | 13.52 ± 1.13 | 11.17 ± 0.79 | | 10.13 ± 1.13 | |  | -2.35 ± 0.90 | | -3.39 ± 1.08 |  |  | |  |
| *p* value |  |  | |  | |  |  | |  |  | .965 | | .831 |
| B-line | .160 | .463 | | .707 | |  | .434 | | .254 |  |  | |  |
| C-line | .209 | .060 | | .266 | |  | .942 | | .342 |  |  | |  |
| D-line | .128 | .347 | | .495 | |  | .291 | | .114 |  |  | |  |

**Table S1. 3D digital analysis for measuring horizontal width and volume changes (Mean ± SD)**

B-line, line drawn 1 mm below the A-line as a reference; C-line, line drawn 3 mm below the A-line; D-line, line drawn 5 mm below the A-line

DBBM: Bio-Oss^®^, membrane: Bio-Gide^®^, matrix: Collagen graft 2^®^

*: Significant different at the same observation period. ± Statistical significance level was 5%, *p* < .05
